# Associations of Waist Circumference, Socioeconomic, Environmental, and Behavioral Factors with Chronic Kidney Disease in Normal Weight, Overweight, and Obese People

**DOI:** 10.3390/ijerph16245093

**Published:** 2019-12-13

**Authors:** Tuyen Van Duong, Pei-Yu Wu, Evelyn Yang, Yuh-Feng Lin, Hung-Yi Chiou, Shwu-Huey Yang

**Affiliations:** 1 School of Nutrition and Health Sciences, Taipei Medical University, Taipei 11031, Taiwan; duongtuyenvna@gmail.com (T.V.D.); d507098001@tmu.edu.tw (P.-Y.W.); 2 Department of Obstetrics and Gynecology, Chung Shan Medical University Hospital, Taichung City 40201, Taiwan; evelyn.yang@hotmail.com; 3 International Master/Ph.D. Program in Medicine, College of Medicine, Taipei Medical University, Taipei 11031, Taiwan; linyfmd@tmu.edu.tw; 4 Division of Nephrology, Department of Internal Medicine, Taipei Medical University−Shuang Ho Hospital, New Taipei 23561, Taiwan; 5 School of Public Health, College of Public Health, Taipei Medical University, Taipei 11031, Taiwan; hychiou@tmu.edu.tw; 6 Health and Clinical Research Data Center, Data Center, Taipei Medical University, Taipei 11031, Taiwan; 7 Master Program in Applied Molecular Epidemiology, Taipei Medical University, Taipei 11031, Taiwan; 8 Research Center of Geriatric Nutrition, Taipei Medical University, Taipei 11031, Taiwan; 9 Nutrition Research Center, Taipei Medical University Hospital, Taipei 11031, Taiwan

**Keywords:** chronic kidney disease, overweight and obesity, case-control, hypertension, diabetes, socioeconomic, exposing to animal, smoking, plain water, Taiwan

## Abstract

*Background:* Chronic kidney disease (CKD) places a heavy burden on the healthcare system worldwide. The risk factors may vary by body adiposity. We aimed to investigate the associations of socioeconomic, environmental, and behavioral factors with CKD in different groups of body mass indexes (BMI). *Methods:* A case-control study was conducted in 3280 participants (1048 CKD and 2232 non-CKD) from seven hospitals and nearby communities from May 2012 to August 2015. Personal characteristics, anthropometrics, environmental exposures, and health−related behaviors were assessed using a structured questionnaire. The logistic regression models were utilized for analysis. *Results:* Older age (odd ratio, OR = 2.85; *p* < 0.001), being men (OR = 4.23; *p* < 0.001), smoking (OR = 3.36; *p* < 0.001), stable income (OR = 0.33; *p* < 0.001), higher education (OR = 0.37~0.38; *p* < 0.001), and daily adequate water intake (OR = 0.64; *p* = 0.010) were associated with CKD in normal weight people. Older age (OR = 2.49; *p* < 0.001), being men (OR = 3.36; *p* < 0.001), education (OR = 0.44, *p* = 0.004), hypertension (OR = 2.93; *p*<0.001), diabetes (OR = 1.83; *p* = 0.004), and using traditional Chinese medicine (OR = 2.03, *p* = 0.014) were associated with CKD in overweight people. Older age (OR = 2.71; *p* < 0.001), being men (OR = 2.69; *p* < 0.001), hypertension (OR = 2.93; *p* < 0.001), diabetes (OR = 1.94; *p* = 0.001) were associated with CKD in obese people. *Conclusions:* The associated factors of CKD varied by different groups of BMI. These findings may help to develop potential interventions to manage CKD.

## 1. Introduction

Chronic kidney disease (CKD) is a major public health issue and significantly contributes to morbidity and mortality globally [1]. CKD places a heavy burden on the healthcare system in every country [2]. The global prevalence of CKD was 10–15% in the general populations [3,4,5], and about 36% in high-risk populations [5]. Taiwan experiences a high prevalence of CKD [6,7,8], and is one of leading countries with highest prevalence of end-stage renal disease [9]. Moreover, awareness of the disease and its risk factors is low in general populations in 12 countries from six world regions [5], and in Taiwan as well [7,10].

It is the consensus that the optimal management of CKD includes cardiovascular risk reduction, treatment of albuminuria, avoidance of potential nephrotoxins, and adjustments to drug dosing [11]. However, factors like socioeconomic, environmental, and behavioral factors have been believed to be important in management of CKD, but have not yet been delineated. From an international perspective, a multidisciplinary, multi−sector, and multifaceted action plan is needed to develop a strategy to prevent and manage CKD in order to combat its burden and complications [4,12]. In Taiwan, the multidisciplinary care helps to slow down the renal progression and improve survival rate [13]. In addition, the risk factors of CKD in people with normal weight, overweight, and obese might be varied. Therefore, we aimed to explore the association between personal characteristics, socioeconomic, environmental factors, and health-related behaviors with CKD in normal, overweight, and obese people.

## 2. Methods

### 2.1. Study Design and Settings

A case-control study was conducted in seven hospitals and the communities nearby from May 2012 to August 2015. Hospitals selected for study included Tri-Service General Hospital (TSGH), Cardinal Tien Hospital (CTH), Shuang Ho Hospital (SHH), China Medical University Hospital (CMUH), Kaohsiung Medical University Hospital (KMUH), Changhua Christian Hospital (CCH), Kaohsiung Chang Gung Memorial Hospital (KCGMH).

### 2.2. Sampling and Sample Size

**Figure 1:**
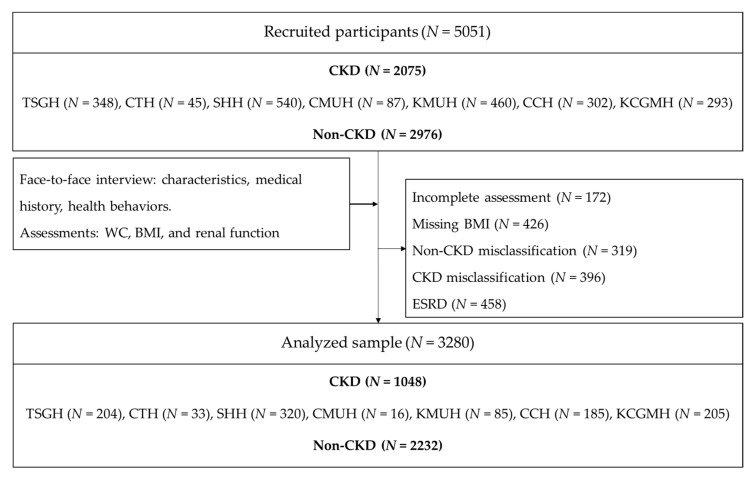
Study flow chart. TSGH, Tri-Service General Hospital; CTH, Cardinal Tien Hospital; SHH, Shuang Ho Hospital; CMUH, China Medical University Hospital; KMUH, Kaohsiung Medical University Hospital; CCH, Changhua Christian Hospital; KCGMH, Kaohsiung Chang Gung Memorial Hospital; WC, waist circumference; BMI, body mass index; CKD, chronic kidney disease; ESRD, end stage renal disease.

Participants recruited were Taiwanese citizens aged 18 years or older, who understand Mandarin Chinese. We excluded participants who were diagnosed as CKD stage 5 or end-stage renal disease (ESRD), or who lacked renal function parameters. Cases were patients with chronic kidney disease stage 1 to stage 4. Controls were non-CKD patients, relatives of CKD patients, healthy people who underwent annual health checks, and in the communities around the selected hospitals. The final sample for analysis was 3280 participants with *N* = 1048 cases, and *N* = 2232 controls (Figure 1).

### 2.3. Instruments and Assessments

*Participants’ characteristics:* Participants were asked to provide their age (years), gender (male vs. female), blood type (O, A, B, AB), marital status (single, married/cohabited, separated/divorced/widow), education attainment (elementary and below, junior high school, senior high school, college/university and above), occupation (unemployed, employed), monthly household income (unstable income, stable income), and medical history (hypertension, diabetes mellitus, hyperlipidemia).

*Anthropometrics:* Body weight, height, and waist circumference (WC) were measured. Abdominal obesity was defined as WC ≥ 90 cm for men and WC ≥ 80 cm for women [14,15]. Body mass index (BMI) was calculated as weight (kg)/[height (m)]^2^. BMI was classified into normal weight (BMI < 24.0 kg/m^2^), overweight (24.0 kg/m^2^ ≤ BMI < 27.0 kg/m^2^), and obese (BMI ≥ 27.0 kg/m^2^) [16].

*Environmental exposures:* Participants were asked to provide information regarding past expose to environmental risk factors such as pesticides, animals (keeping pets or poultry, slaughtering or selling chicken, ducks, etc.), chemicals (asbestos, heavy metal lead, mercury, polychlorinated biphenyls, etc.), and organic solvents or compounds (e.g., gasoline, derivatives, plywood adhesives, paints, methane, rosin, etc.).

*Health-related behaviors:* The behaviors assessed included daily intake of water (<1.5 L/day vs. ≥1.5 L/day), using traditional Chinese medicine products (yes vs. no), smoking tobacco (yes vs. no), drinking alcohol (yes vs. no), and chewing betel nut (yes vs. no). Betel quid and areca nut, called betel nut, is widely used as plant chewing gum in Asian communities [17]. It was summarized that betel nut chewing was associated with increased CKD risk [18]. To evaluate physical activity level, the short version of the International Physical Activity Questionnaire (IPAQ), a widely used method, was utilized [19]. Patients were asked to provide information on their time spent (days per week, and minutes per day) on different levels of physical intensity (vigorous, moderate, walking, and sitting). The overall physical activity score was given by calculating the sum of minutes spent on activities at different levels of vigorous, moderate, walking, and sitting over the last seven days multiplied by 8.0, 4.0, and 3.3, 1.0, respectively [19]. The common method using metabolic equivalent task scored in minute per week (named as MET-min/wk) was used to represent the physical activity [20].

*CKD classification:* Kidney function parameters were analyzed in the hospital laboratories and the results were shown in medical records. The estimated glomerular filtration rate (eGFR, mL/min/1.73 m^2^) was calculated using the Chronic Kidney Disease Epidemiology Collaboration (CKD−EPI) equation [21]. eGFR = 141 × min (SCr/κ, 1)^α^ × max (SCr/κ, 1)^−1.209^ × 0.993^Age^ × (1.018 if female) × (1.159 if black)], where κ = 0.7 (female) and 0.9 (male), α = −0.329 (female) and −0.411 (male), serum creatinine (SCr, mg/dL), min indicated the minimum of SCr/κ or 1, and max indicated the maximum of SCr/κ or 1.

Nurses then checked to confirm the stage of CKD for each participant. CKD was classified into different stages: *Stage 1* as an eGFR ≥ 90.0 mL/min/1.73 m^2^, and the presence of kidney damage (e.g., proteinuria dipsticks ≥1+, urine protein-to-creatinine ratio [UPCR] ≥ 150, or urine albumin-to-creatinine ratio [UACR] ≥ 30); *Stage 2* as an eGFR = 60.0–89.9 mL/min/1.73 m^2^, and the presence of kidney damage (e.g., proteinuria dipsticks ≥ 1+, UPCR ≥ 150, or UACR ≥ 30); *Stage 3a* as an eGFR = 45.0–59.9 mL/min/1.73 m^2^; *Stage 3b* as an eGFR = 30.0–44.9 mL/min/1.73 m^2^; *Stage 4* as an eGFR = 15.0–29.9 mL/min/1.73 m^2^; and *Stage 5* as an eGFR < 15.0 mL/min/1.73 m^2^ [22]. Participants were defined as non-CKD only if they had an eGFR ≥ 60.0 mL/min/1.73 m^2^, and did not have any evidence of kidney damage [22].

### 2.4. Data Collection Procedures

Interviewers were seven research assistants from seven hospitals. They received 4 h of training for data collection. Patients and healthy people participated in the study voluntarily and were checked by research assistants for eligibility. The face-to-face interviews were conducted in hospitals or in community activity stations. After interviews, blood specimens were collected by registered nurses and then were analyzed in hospital laboratories to determine renal function parameters. The same laboratory criteria and protocol were adopted for all hospitals involved. The diagnosis of CKD was based on the hospital laboratory results. All the misclassification of patients from interviews or lack of renal function parameters were excluded from final sample. The study procedure was recapped in Figure 1.

### 2.5. Ethical Approvals

The study protocol was approved by the ethical committee of Taipei Medical University Joint Institutional Review Board of Taipei Medical University (TMU-JIRB No. 201204036). All participants signed the written informed consent form before their participation.

### 2.6. Data Analysis

The studied variables were described as mean and standard deviation, frequency and percentage, median and interquartile range. The independent-samples T-test and one-way ANOVA test, and the Mann Whitney test were used to compare the distribution of studied variables between non-CKD and CKD groups, appropriately. Finally, the bivariate logistic regression model was used to examine the associations of participants’ characteristics, medical history, health-related behaviors with CKD in the overall sample, in the group with normal weight, and in the overweight/obese group. Variables selected into the multivariate logistic regression model were those that showed the association with CKD at *p* < 0.20 from the bivariate model [23]. In order to avoid multicollinearity in the multivariate analysis, the Spearman correlation was utilized to check the correlations between selected variables. Analysis was conducted using the IBM SPSS software version 20.0 for Windows (IBM Corp., Armonk, NY, USA). The significant level was set at *p*-value < 0.05.

## 3. Results

The average age of the study population was 60.5 ± 13.2 years, 47.3% were men, 29.2% were overweight, and 24.6% were obesity. The prevalence of CKD was varied by different categories of age, gender, blood type, BMI, WC, marital status, education, occupation, household income, history of diseases, exposure to pesticide and animal, daily intake of plain water, smoking, chewing betel nut, and physical activities (Table 1).

In the overall sample, results of simple regression analysis showed that age, gender, blood type, BMI, WC, marital status, education, occupation, income, hypertension, diabetes, hyperlipidemia, exposure to pesticide and animal, daily intake of plain water, smoking, and chewing betel nut were significantly associated with CKD (Table 2). Furthermore, the correlations between variables (associated with CKD at *p* < 0.20) were examined. Moderate correlations were found between WC and BMI (*r* = 0.52), age and education (*r* = −0.31), gender and smoking (*r* = 0.48), smoking and betel nut (*r* = 0.47) (Table S1). Age, gender, WC, betel nut, and other factors were analyzed in the multivariate analysis. We found that factors showing significantly positive association with CKD were age ≥65 years (odd ratio, OR, 2.43; 95% confidence interval, 95% CI, 1.93–3.07; *p* < 0.001), men (OR, 2.96; 95% CI, 2.32–3.78; *p* < 0.001), abdominal obesity (OR, 1.52; 95% CI, 1.21–1.91; *p* < 0.001), hypertension (OR, 4.35; 95% CI, 3.46–5.47; *p* < 0.001), diabetes mellitus (OR, 1.36; 95% CI, 1.07–1.72; *p* = 0.011), and exposure to animals (OR, 1.37; 95% CI, 1.04–1.80; *p* = 0.027; Table 3). Factors showing the negative association with CKD were married/cohabited status (OR, 0.60; 95% CI, 0.38–0.96; *p* = 0.031), stable income (OR, 0.61; 95% CI, 0.48–0.76; *p* < 0.001), and drinking ≥ 1.5 L/day plain water (OR, 0.69; 95% CI, 0.56–0.87; *p* = 0.001; Table 3).

In the normal weight group, results of simple regression analysis showed that age, gender, WC, marital status, education, occupation, income, hypertension, diabetes, hyperlipidemia, exposure to animals, daily intake of plain water, and smoking were significantly associated with CKD (Table 2). Moderate correlations were existed between age and education (*r* = −0.32), age and history of hypertension (*r* = −0.33), gender and smoking (*r* = 0.46), smoking and chewing betel nut (*r* = 0.44) (Table S1). Age, gender, chewing betel nut, and other factors were analyzed in the multivariate analysis. We found that the factors significantly positively associated with CKD were age ≥65 years (OR, 2.85; 95% CI, 2.00–4.06; *p* < 0.001), men (OR, 4.23; 95% CI, 2.88–6.19; *p* < 0.001). The factors showing the negative association with CKD were stable income (OR, 0.33; 95% CI, 0.23–0.47; *p* < 0.001), and drinking ≥1.5 L/day of plain water (OR, 0.64; 95% CI, 0.46–0.90; *p* = 0.010; Table 3).

In the overweight group, results of simple regression analysis showed that age, gender, WC, marital status, education, occupation, income, hypertension, diabetes, hyperlipidemia, and smoking were significantly associated with CKD (Table 2). Moderate correlations were existed between gender and smoking (*r* = 0.46), smoking and chewing betel nut (*r* = 0.45) (Table S1). Gender, chewing betel nut, and other factors were analyzed in the multivariate analysis. We found that the factors showing significantly positive association with CKD were age ≥65 years (OR, 2.49; 95% CI, 1.62–3.82; *p* < 0.001), being men (OR, 3.36; 95% CI, 2.07–5.45; *p* < 0.001), hypertension (OR, 2.93; 95% CI, 1.95–4.40; *p* < 0.001), diabetes (OR, 1.83; 95% CI, 1.21–2.78; *p* = 0.004), using traditional Chinese medicine (TCM) products (OR, 2.03; 95% CI, 1.15–3.56; *p* = 0.014). Factors showing negative associations with CKD was education at vocational/college/ or university level (OR, 0.44; 95% CI, 0.25–0.77; *p* = 0.004; Table 3).

In the obese group, results of simple regression analysis showed that age, gender, blood type, WC, education, income, history of hypertension, diabetes, hyperlipidemia, exposure to pesticides, daily intake of plain water, and smoking were significantly associated with CKD (Table 2). Moderate correlations were observed between age and education (*r* = −0.34), gender and smoking (*r* = 0.51), gender and chewing betel nut (*r* = 0.33), smoking and chewing betel nut (*r* = 0.51; Table S1). Age, gender, and other factors were analyzed in the multivariate model. We found that the factors showing significantly positive association with CKD were age ≥65 years (OR, 2.71; 95% CI, 1.81–4.05; *p* < 0.001), being men (OR, 2.69; 95% CI, 1.75–4.16; *p* < 0.001), hypertension (OR, 2.93; 95% CI, 1.96–4.37; *p* < 0.001), diabetes (OR, 1.94; 95% CI, 1.29–2.91; *p* = 0.001; Table 3).

## 4. Discussion

The current study showed that people aged ≥65 years had a higher likelihood of CKD. Age was found to be a risk factor of CKD in previous studies [24,25,26]. Additionally, our study showed that men have a higher probability of having CKD than women. Note that, across all regions, it has been shown that men were closely associated with the occurrence of CKD [27]; by contrast, in other parts of the world such as in China, Switzerland, and Iran, women had a higher risk of CKD than men [24,28,29]. In China, authors analyzed people with eGFR ≥ 60.0 mL/min/1.73 m^2^ [24], while we analyzed people with CKD at stage 1 to stage 4, the different selection criteria might affect the results. In Switzerland and Iran, the different genetic background might contribute to this contradiction [30]. Based on this evidence, we conclude that gender should be taken into account when developing strategical approaches to prevent and treat CKD and its complications [31]. Moreover, we found that people with higher education and stable income had a lower likelihood of having CKD, a finding consistent with previous studies showing that low socioeconomic status (low education, occupation, and income) was strongly associated with a higher risk of having CKD [32,33].

In the multivariate model, being married or cohabited had a lower likelihood of having CKD as compared with being unmarried in the overall sample. This could be explained by the previous evidence that being unmarried or living alone was associated with higher systolic blood pressure [34], a high prevalence of hypertension [35], and lower hypertension awareness and control rate [36,37]. Hypertension has been proven to be a strong predictor of CKD [24]. In addition, married people had lower prevalence of diabetes and smoking [38]. Diabetes [39,40], and smoking [26] were recognized as important predictors of CKD. Taken together, married or cohabited people had lower blood pressure, higher hypertension awareness and control, and lower prevalence of diabetes and smoking, which, in turn, led to a lower risk of CKD as compared with unmarried people.

Our study illustrated that people with hypertension and diabetes had a higher likelihood of CKD. It has been proven that hypertension is a cause and complication of CKD [24,41,42,43]. In addition, diabetes was one of the most common causes of CKD [39,40,43]. On the other hand, insulin resistance was independently associated with cardiovascular risks in ESRD patients [44]. Therefore, there is a consensus that better management of hypertension, and diabetes is required to improve CKD patient care [45,46,47].

Our data showed that, general overweight and obesity was positively associated with a higher likelihood of CKD in the overall sample. The previous evidence showed that obesity measured by different adiposity indices (percentage body fat, visceral fat index, body mass index, waist circumference, and waist-to-height ratio) was significantly associated with increased risk of having CKD [48]. In addition, results of bivariate analysis showed that elevated WC was significantly associated with higher prevalence of CKD in the overall sample, in overweight, and in obese individuals as well. This was partly supported by the evidence that the effect of obesity in triggering CKD was only significant in the obese population [49].

Our study showed that people exposed to animals had a higher CKD occurrence. This might be explained by environmental factors also being a common cause of CKD [39]. In addition, people who had a daily intake of plain water ≥1.5 L of plain water had a lower likelihood of developing CKD. Water is an essential nutrient for body hydration [50]. A previous study showed that increasing daily water consumption slowed down the progression of CKD [51]. Moreover, overweight people using traditional Chinese medicine (TCM) products had a higher rate of CKD as compared to those did not use. A number of studies found the adverse effect of TCM on CKD [52]. However, the evidence is insufficient, the practice of TCM in varied, rigorous, well−designed studies is required to provide evidence-based practices to improve the safety and efficacy [52].

Current smoking was associated with a higher prevalence of CKD in the current study. Smoking has been recognized as a risk factor for CKD in both men and women [26], and smoking increased the risk for cardiovascular events, ESRD, and mortality in CKD patients [53,54]. Alcohol consumption was not associated with CKD in our study. This could be explained that the quality of alcohol was not measured in the current study. The previous study showed that alcohol intake was associated with incident CKD [55]. It is suggested that limited alcohol consumption could minimize the health risks [56] and delay CKD progression [57].

This study had some limitations. Firstly, a causal relationship cannot be drawn from a cross-sectional design. However, the findings from this study might be useful for future epidemiological studies or public health programs. Secondly, study indicators were subjectively assessed using a structured questionnaire. However, considering that (1) interviewers were well-trained for data collection, and (2) Taiwanese people have high self-awareness in personal health under universal health coverage policy. These factors may reduce the interview bias. Future studies with a larger sample and better study design are suggested to comprehensively explore the risk factors and assure the causality.

## 5. Conclusions

In normal weight people, older age, being men, and smoking were associated with a higher prevalence of CKD, while stable income and drinking enough daily plain water were associated with a lower prevalence of CKD. In overweight people, older age, being men, history of hypertension, diabetes, using TCM products, and smoking were associated with a higher prevalence of CKD, while higher education status was found to be a protective factor of CKD. In obese people, older age, being men, history of hypertension, diabetes, and smoking were associated with a higher rate of CKD. The evidence obtained from this study may help to improve multifaceted strategical interventions to prevent and manage CKD.

## Figures and Tables

**Table 1:** Participants’ characteristics.

Variables	Total (*N* = 3280)	Non−CKD (*N* = 2232)	CKD (*N* = 1048)	
	n (%)	n (%)	n (%)	*p*-value *
Age, year, mean ± SD	60.5 ± 13.2	57.6 ± 12.2	66.7 ± 13.2	<0.001
Age groups				<0.001
<65	2022 (61.6)	1599 (71.6)	423 (40.4)	
≥65	1258 (38.4)	633 (28.4)	625 (59.6)	
Gender				<0.001
Women	1728 (52.7)	1361 (61.0)	367 (35.0)	
Men	1552 (47.3)	871 (39.0)	681 (65.0)	
Blood type				0.002
O	1247 (44.5)	853 (44.4)	394 (44.7)	
A	758 (27.1)	534 (27.8)	224 (25.4)	
B	626 (22.3)	400 (20.8)	226 (25.7)	
AB	170 (6.1)	133 (6.9)	37 (4.2)	
BMI, kg/m^2^				<0.001
BMI < 24.0	1516 (46.2)	1128 (50.5)	388 (37.0)	
24.0 ≤ BMI < 27.0	956 (29.2)	647 (29.0)	309 (29.5)	
BMI ≥ 27.0	808 (24.6)	457 (20.5)	351 (33.5)	
WC, cm				<0.001
Normal	1565 (55.5)	1193 (60.6)	372 (43.8)	
Abdominal obesity	1253 (44.5)	776 (39.4)	477 (56.2)	
Marital status				<0.001
Single	241 (7.4)	186 (8.4)	55 (5.3)	
Married/Cohabited	2667 (81.7)	1822 (82.0)	845 (81.0)	
Divorce/separate/widow	356 (10.9)	213 (9.6)	143 (13.7)	
Education				<0.001
Elementary and below	958 (29.3)	533 (24.0)	425 (40.6)	
Secondary school	423 (12.9)	266 (12.0)	157 (15.0)	
High school	845 (25.9)	619 (27.9)	226 (21.6)	
Vocational/College/University	1042 (31.9)	803 (36.2)	239 (22.8)	
Occupation				<0.001
Unemployed	209 (6.4)	105 (4.7)	104 (10.1)	
Employed	3034 (93.6)	2111 (95.3)	923 (89.9)	
Monthly household income				<0.001
Unstable income	1177 (37.4)	642 (30.5)	535 (51.5)	
Stable income	1970 (62.6)	1466 (69.5)	504 (48.5)	
Medical history				
Hypertension				<0.001
No	1934 (60.4)	1620 (75.1)	314 (30.1)	
Yes	1267 (39.6)	538 (24.9)	729 (69.9)	
Diabetes				<0.001
No	2328 (72.7)	1698 (78.7)	630 (60.4)	
Yes	873 (27.3)	460 (21.3)	413 (39.6)	
Hyperlipidemia				<0.001
No	2618 (81.8)	1842 (85.4)	776 (74.4)	
Yes	583 (18.2)	316 (14.6)	267 (25.6)	
Environmental factors				
Exposure to pesticides				0.007
No	2922 (96.4)	1972 (97.0)	950 (95.1)	
Yes	109 (3.6)	60 (3.0)	49 (4.9)	
Exposure to animals				0.017
No	2429 (80.1)	1653 (81.3)	776 (77.7)	
Yes	602 (19.9)	379 (18.7)	223 (22.3)	
Exposure to chemicals				0.153
No	2960 (97.7)	1990 (97.9)	970 (97.1)	
Yes	71 (2.3)	42 (2.1)	29 (2.9)	
Exposure to solvents				0.120
No	2901 (95.7)	1953 (96.1)	948 (94.9)	
Yes	130 (4.3)	79 (3.9)	51 (5.1)	
Health-related behaviors				
Daily intake of plain water				<0.001
<1.5 L/day	1604 (48.9)	1020 (45.7)	584 (55.7)	
≥1.5 L/day	1676 (51.1)	1212 (54.3)	464 (44.3)	
Using TCM products				0.195
No	2559 (83.3)	1707 (83.9)	852 (82.1)	
Yes	513 (16.7)	327 (16.1)	186 (17.9)	
Smoking tobacco				<0.001
No	2514 (78.8)	1834 (85.2)	680 (65.4)	
Yes	677 (21.2)	318 (14.8)	359 (34.6)	
Drinking alcohol				0.349
No	2601 (81.8)	1761 (82.2)	840 (80.8)	
Yes	580 (18.2)	381 (17.8)	199 (19.2)	
Chewing betel nut				<0.001
No	2914 (91.9)	1985 (93.2)	929 (89.2)	
Yes	256 (8.1)	144 (6.8)	112 (10.8)	
PA, METs-min/wk	1080.0 (594.0–2034.0)	1116.0 (600.0–2118.0)	1032.0 (594.0–1813.0)	0.041

Abbreviations: CKD, chronic kidney disease; SD, standard deviation; BMI, body mass index; WC, waist circumference; TCM, traditional Chinese medicine; PA−METs, physical activity measured in metabolic equivalents (min/week). * Data were presented as mean ± SD, frequency and percentage, median (interquartile range) and *p*-values were calculated using independent sample *t*-test, Chi-square test, and Mann Whitney test, appropriately.

**Table 2:** The associated factors of CKD in the overall sample, in the groups with normal weight, overweight, and obesity using simple logistic regression models.

Variables	Total (*N* = 3280)		BMI < 24.0 (*N* = 1516)		24.0 ≤ BMI < 27.0 (*N* = 956)		BMI ≥ 27.0 (*N* = 808)	
	OR (95% CI)	*p*	OR (95% CI)	*p*	OR (95% CI)	*p*	OR (95% CI)	*p*
Age groups								
<65	Reference		Reference		Reference		Reference	
≥65	3.73 (3.20–4.35)	<0.001	4.43 (3.47–5.66)	<0.001	4.26 (3.20–5.67)	<0.001	2.77 (2.07–3.71)	<0.001
Gender								
Women	Reference		Reference		Reference		Reference	
Men	2.90 (2.49–3.38)	<0.001	3.52 (2.77–4.48)	<0.001	2.39 (1.80–3.17)	<0.001	2.33 (1.75–3.10)	<0.001
Blood type								
O	Reference		Reference		Reference		Reference	
A	0.91 (0.75–1.11)	0.336	0.96 (0.71–1.29)	0.777	0.85 (0.58–1.23)	0.390	0.93 (0.64–1.36)	0.722
B	1.22 (1.00–1.50)	0.051	0.97 (0.70–1.35)	0.857	1.40 (0.97–2.01)	0.072	1.48 (1.00–2.19)	0.050
AB	0.60 (0.41–0.88)	0.010	0.67 (0.35–1.25)	0.204	0.62 (0.32–1.19)	0.148	0.47 (0.23–0.97)	0.042
BMI, kg/m^2^								
BMI < 24.0	Reference							
24.0 ≤ BMI < 27.0	1.39 (1.16–1.66)	<0.001						
BMI ≥ 27.0	2.23 (1.86–2.68)	<0.001						
WC, cm								
Normal	Reference		Reference		Reference		Reference	
Abdominal obesity	1.97 (1.68–2.32)	< 0.001	1.45 (1.05–2.01)	0.026	1.52 (1.12–2.05)	0.007	1.76 (1.14–2.72)	0.011
Marital status								
Single	Reference		Reference		Reference		Reference	
Married/Cohabited	1.57 (1.15–2.14)	0.005	2.12 (1.28–3.49)	0.003	1.94 (0.92–4.08)	0.081	0.91 (0.53–1.54)	0.716
Divorce/separate/widow	2.27 (1.57–3.28)	<0.001	2.90 (1.62–5.18)	<0.001	2.74 (1.19–6.29)	0.017	1.59 (0.82–3.08)	0.168
Education								
Elementary and below	Reference		Reference		Reference		Reference	
Secondary school	0.74 (0.59–0.94)	0.012	0.76 (0.52–1.11)	0.157	0.73 (0.46–1.14)	0.161	0.73 (0.48–1.11)	0.137
High school	0.46 (0.38–0.56)	<0.001	0.38 (0.28–0.53)	<0.001	0.59 (0.41–0.84)	0.004	0.54 (0.38–0.79)	0.001
Vocational/College/University	0.37 (0.31–0.45)	<0.001	0.37 (0.27–0.50)	<0.001	0.44 (0.31–0.62)	<0.001	0.42 (0.29–0.61)	<0.001
Occupation								
Unemployed	Reference		Reference		Reference		Reference	
Employed	0.44 (0.33–0.59)	<0.001	0.40 (0.26–0.62)	<0.001	0.36 (0.21–0.60)	<0.001	0.67 (0.40–1.14)	0.138
Monthly household income								
Unstable income	Reference		Reference		Reference		Reference	
Stable income	0.41 (0.35–0.48)	<0.001	0.35 (0.27–0.45)	<0.001	0.55 (0.42–0.73)	<0.001	0.45 (0.34–0.60)	<0.001
Medical history								
Hypertension								
No	Reference		Reference		Reference		Reference	
Yes	6.99 (5.93–8.24)	<0.001	8.98 (6.91–11.67)	<0.001	4.14 (3.10–5.54)	<0.001	7.12 (5.11–9.91)	<0.001
Diabetes								
No	Reference		Reference		Reference		Reference	
Yes	2.42 (2.06–2.84)	<0.001	2.42 (1.85–3.18)	<0.001	2.12 (1.59–2.83)	<0.001	2.07 (1.54–2.77)	<0.001
Hyperlipidemia								
No	Reference		Reference		Reference		Reference	
Yes	2.01 (1.67–2.41)	<0.001	1.89 (1.40–2.55)	<0.001	1.55 (1.09–2.20)	0.015	2.20 (1.58–3.05)	<0.001
Environmental factors								
Exposure to pesticides								
No	Reference		Reference		Reference		Reference	
Yes	1.70 (1.15–2.49)	0.007	1.19 (0.58–2.44)	0.630	1.28 (0.59–2.78)	0.527	2.06 (1.10–3.85)	0.023
Exposure to animals								
No	Reference		Reference		Reference		Reference	
Yes	1.25 (1.04–1.51)	0.017	1.35 (1.01–1.81)	0.043	1.10 (0.78–1.56)	0.588	1.21 (0.85–1.71)	0.291
Exposure to Chemicals								
No	Reference		Reference		Reference		Reference	
Yes	1.42 (0.88–2.29)	0.155	1.05 (0.49–2.29)	0.896	1.59 (0.62–4.07)	0.335	1.90 (0.77–4.70)	0.165
Exposure to solvents								
No	Reference		Reference		Reference		Reference	
Yes	1.33 (0.93–1.91)	0.121	1.39 (0.79–2.43)	0.254	1.67 (0.83–3.37)	0.150	0.96 (0.50–1.84)	0.902
Health-related behaviors								
Daily intake of plain water								
<1.5 L/day	Reference		Reference		Reference		Reference	
≥1.5 L/day	0.67 (0.58–0.78)	<0.001	0.57 (0.45–0.72)	<0.001	0.80 (0.61–1.05)	0.115	0.64 (0.48–0.84)	0.002
Using TCM products								
No	Reference		Reference		Reference		Reference	
Yes	1.14 (0.94–1.39)	0.196	0.94 (0.69–1.29)	0.701	1.41 (0.98–2.03)	0.064	1.24 (0.85–1.81)	0.271
Smoking tobacco								
No	Reference		Reference		Reference		Reference	
Yes	3.05 (2.56–3.63)	<0.001	3.36 (2.53–4.47)	<0.001	2.70 (1.96–3.70)	<0.001	2.51 (1.82–3.45)	<0.001
Drinking alcohol								
No	Reference		Reference		Reference		Reference	
Yes	1.10 (0.91–1.32)	0.350	1.25 (0.92–1.70)	0.157	0.97 (0.68–1.37)	0.841	0.91 (0.64–1.28)	0.587
Chewing betel nut								
No	Reference		Reference		Reference		Reference	
Yes	1.66 (1.28–2.15)	<0.001	1.53 (0.95–2.48)	0.083	1.44 (0.91–2.26)	0.120	1.52 (0.99–2.35)	0.057
PA, METs-min/wk								
1st tertile	Reference		Reference		Reference		Reference	
2nd tertile	0.93 (0.77–1.13)	0.464	0.82 (0.60–1.11)	0.197	0.97 (0.68–1.38)	0.872	1.14 (0.78–1.65)	0.501
3rd tertile	1.01 (0.84–1.23)	0.885	1.00 (0.74–1.35)	0.993	1.08 (0.75–1.55)	0.678	1.12 (0.76–1.65)	0.564

Abbreviations: CKD, chronic kidney disease; BMI, body mass index; OR, odds ratio; CI, confidence interval; WC, waist circumference; TCM, traditional Chinese medicine; PA, physical activity; METs, metabolic equivalents (min/week).

**Table 3:** The associated factors of CKD in overall sample, in the groups with normal weight, overweight, and obesity using multiple logistic regression models.

Variales	Total (*N* = 3280)		BMI < 24.0 (*N* = 1516)		24.0 ≤ BMI < 27.0 (*N* = 956)		BMI ≥ 27.0 (*N* = 808)	
	OR (95% CI)	*p*	OR (95% CI)	*p*	OR (95% CI)	*p*	OR (95% CI)	*p*
Age groups								
<65	Reference		Reference		Reference		Reference	
≥65	2.43 (1.93–3.07)	<0.001	2.85 (2.00–4.06)	<0.001	2.49 (1.62–3.82)	<0.001	2.71 (1.81–4.05)	<0.001
Gender								
Women	Reference		Reference		Reference		Reference	
Men	2.96 (2.32–3.78)	<0.001	4.23 (2.88–6.19)	<0.001	3.36 (2.07–5.45)	<0.001	2.69 (1.75–4.16)	<0.001
Blood type								
O	Reference				Reference		Reference	
A	0.96 (0.74–1.26)	0.775			0.85 (0.51–1.40)	0.517	0.80 (0.49–1.31)	0.379
B	1.17 (0.89–1.55)	0.264			1.53 (0.93–2.51)	0.094	1.45 (0.89–2.35)	0.137
AB	0.71 (0.43–1.16)	0.170			0.65 (0.28–1.50)	0.311	0.64 (0.28–1.45)	0.284
WC, cm								
Normal	Reference		Reference		Reference		Reference	
Abdominal obesity	1.52 (1.21–1.91)	<0.001	1.31 (0.83–2.06)	0.243	1.32 (0.86–2.03)	0.199	1.34 (0.88–2.03)	0.172
Marital status								
Single	Reference		Reference		Reference		Reference	
Married/Cohabited	0.60 (0.38–0.96)	0.031	0.98 (0.46–2.07)	0.948	0.74 (0.27–2.05)	0.560	0.85 (0.31–2.32)	0.754
Divorce/separate/widow	0.86 (0.49–1.50)	0.591	1.44 (0.60–3.45)	0.413	0.91 (0.28–2.90)	0.869	1.15 (0.37–3.59)	0.806
Education								
Elementary and below					Reference			
Secondary school					0.68 (0.36–1.27)	0.226		
High school					0.63 (0.36–1.12)	0.119		
Vocational/College/University					0.44 (0.25–0.77)	0.004		
Occupation								
Unemployed	Reference		Reference		Reference		Reference	
Employed	0.71 (0.45–1.12)	0.142	0.63 (0.31–1.30)	0.212	0.66 (0.30–1.46)	0.306	0.64 (0.30–1.38)	0.257
Monthly household income								
Unstable income	Reference		Reference		Reference		Reference	
Stable income	0.61 (0.48–0.76)	<0.001	0.33 (0.23–0.47)	<0.001	0.96 (0.63–1.46)	0.839	0.82 (0.55–1.22)	0.321
Medical history								
Hypertension								
No	Reference				Reference		Reference	
Yes	4.35 (3.46–5.47)	<0.001			2.93 (1.95–4.40)	<0.001	2.93 (1.96–4.37)	<0.001
Diabetes								
No	Reference		Reference		Reference		Reference	
Yes	1.36 (1.07–1.72)	0.011	1.05 (0.71–1.55)	0.816	1.83 (1.21–2.78)	0.004	1.94 (1.29–2.91)	0.001
Hyperlipidemia								
No	Reference		Reference		Reference		Reference	
Yes	1.29 (0.98–1.69)	0.070	1.44 (0.92–2.25)	0.111	1.16 (0.68–1.97)	0.590	1.09 (0.64–1.83)	0.759
Environmental factors								
Exposure to pesticides								
No	Reference						Reference	
Yes	1.38 (0.76–2.51)	0.287					1.22 (0.44–3.37)	0.702
Exposure to animals								
No	Reference		Reference					
Yes	1.37 (1.04–1.80)	0.027	1.48 (0.98–2.24)	0.060				
Exposure to Chemicals								
No	Reference						Reference	
Yes	1.07 (0.51–2.25)	0.857					1.57 (0.37–6.73)	0.542
Exposure to solvents								
No	Reference				Reference			
Yes	1.21 (0.70–2.11)	0.490			1.06 (0.35–3.23)	0.921		
Health-related behaviors								
Daily intake of plain water								
<1.5 L/day	Reference		Reference		Reference		Reference	
≥1.5 L/day	0.69 (0.56–0.87)	0.001	0.64 (0.46–0.90)	0.010	0.72 (0.48–1.07)	0.100	0.71 (0.48–1.05)	0.087
Using TCM products								
No	Reference				Reference			
Yes	1.35 (0.99–1.83)	0.059			2.03 (1.15–3.56)	0.014		
Drinking alcohol								
No			Reference		Reference			
Yes			0.93 (0.59–1.47)	0.750	0.89 (0.52–1.51)	0.665		
Chewing betel nut								
No	Reference		Reference		Reference			
Yes	0.84 (0.57–1.24)	0.372	0.97 (0.49–1.94)	0.936	0.92 (0.46–1.86)	0.826		
PA, METs-min/wk								
1st tertile			Reference					
2nd tertile			0.88 (0.57–1.35)	0.566				
3rd tertile			1.08 (0.72–1.63)	0.708				

Abbreviations: CKD, chronic kidney disease; BMI, body mass index; OR, odds ratio; CI, confidence interval; WC, waist circumference; TCM, traditional Chinese medicine; PA, physical activity; METs, metabolic equivalents (min/week).

## Supplementary Materials

The following are available online at https://www.mdpi.com/1660-4601/16/24/5093/s1, Table S1. The Spearman correlation among the variables associated with chronic kidney disease at *p* < 0.20.
